# One-Pot Sol–Gel Synthesis of Highly Insulative Hybrid P(AAm-CO-AAc)-Silica Aerogels with Improved Mechanical and Thermal Properties

**DOI:** 10.3390/gels9080651

**Published:** 2023-08-12

**Authors:** Akshay A. Ransing, Rushikesh P. Dhavale, Vinayak G. Parale, Uzma K. H. Bangi, Haryeong Choi, Wonjun Lee, Jiseung Kim, Qi Wang, Varsha D. Phadtare, Taehee Kim, Wook Ki Jung, Hyung-Ho Park

**Affiliations:** 1 Department of Materials Science and Engineering, Yonsei University, Seoul 03722, Republic of Korea; akshayransing2@yonsei.ac.kr (A.A.R.); rushi@yonsei.ac.kr (R.P.D.); vinayakparale3@gmail.com (V.G.P.); haryeong@yonsei.ac.kr (H.C.); wonjun_lee@yonsei.ac.kr (W.L.); jiseung_kim@yonsei.ac.kr (J.K.); qwang@yonsei.ac.kr (Q.W.); varshaphadtarev@gmail.com (V.D.P.); taehee-kim@yonsei.ac.kr (T.K.); 2 Department of Physics, School of Physical Sciences, Punyashlok Ahilyadevi Holkar Solapur University, Solapur—Pune National Highway, Solapur 413 255, Maharashtra, India; uzma.phys@gmail.com; 3 Agency for Defense Development (ADD), Daejeon 34146, Republic of Korea; wookki_jung@add.re.kr

**Keywords:** one-pot sol–gel synthesis, hybrid P(AAm-CO-AAc)-silica aerogel, epoxy ring-opening polymerization, thermal stability, mechanical properties

## Abstract

Silica aerogels and their derivatives have outstanding thermal properties with exceptional values in the thermal insulation industry. However, their brittle nature restricts their large-scale commercialization. Thus, enhancing their mechanical strength without affecting their thermal insulating properties is essential. Therefore, for the first time, highly thermally stable poly(acrylamide-co-acrylic acid) partial sodium salt is used as a reinforcing polymer to synthesize hybrid P(AAm-CO-AAc)-silica aerogels via epoxy ring-opening polymerization in the present study. Functional groups in P(AAm-CO-AAc) partial sodium salts, such as CONH_2_ and COOH, acted as nucleophiles for the epoxy ring-opening reaction with (3-glycidyloxypropyl)trimethoxysilane, which resulted in a seven-fold enhancement in mechanical strength compared to that of pristine silica aerogel while maintaining thermal conductivity at less than 30.6 mW/mK and porosity of more than 93.68%. Moreover, the hybrid P(AAm-CO-AAc)-silica aerogel demonstrated improved thermal stability up to 343 °C, owing to the synergetic effect between the P(AAm-CO-AAc) and the silica aerogel, corresponding to the thermal stability and strong covalent bonding among them. These excellent results illustrate that this new synthetic approach for producing hybrid P(AAm-CO-AAc)-silica aerogels is useful for enhancing the mechanical strength of pristine silica aerogel without impairing its thermal insulating property and shows potential as an industrial heat insulation material.

## 1. Introduction

In recent years, the heat insulation industry has paid a great deal of attention to silica aerogels due to their noteworthy properties, such as very low density (0.03–0.1 g/cm^3^), high porosity (90–99%), high specific surface area (100–1000 m^2^/g), and low thermal conductivity (0.02–0.044 W/mK) [1,2,3,4,5]. Moreover, their fascinating properties make them suitable for a wide range of applications in aerospace and other industries, including sensors, building construction, super-insulated windows, and energy storage devices [6,7,8,9,10,11,12]. However, besides the outstanding properties of silica aerogels, their brittle and fragile nature constrains their long-term and practicability for heat insulation. Therefore, to overcome these shortcomings of silica aerogels, researchers are currently focused on enhancing their mechanical strength without affecting their intrinsic properties [13,14,15].

Until now, several methods have been explored to overcome this strengthening problem, such as aging, surface modification, co-precursor methods, and radical polymerization, as well as organic polymers and fibers for synthesizing hybrid or composite-reinforced silica aerogels [16,17,18,19,20,21]. Considering feasible and worthwhile ways to enhance the structural strength of silica aerogel, several researchers have recently tried to focus on synthesizing hybrid organic–inorganic silica aerogels in which different organic or synthetic polymers are crosslinked with the silica aerogel using various crosslinkers. For instance, polyurea, epoxides, polyacrylates, polyimides, and polystyrene polymers have been crosslinked with pristine silica aerogel by using diisocyanates, (3-aminopropyl)triethoxysilane, acrylates, anhydrides, and vinyl trimethoxysilane, among others, as the crosslinker, respectively [13,22,23,24,25,26,27]. Moreover, radical polymerization of alkene and vinyl-functionalized monomers represents another effective reinforcement method based on vinyl trimethoxysilane, vinylmethyldimethoxysiloxane, or vinylmethyldiethoxysiloxane to enhance the mechanical properties and stability of silica aerogel [21,28]. Two different strategies have mainly been used to incorporate organic polymers as structural reinforcing agents into the silica framework. One is the post-gelation washing method, whereby a solid silica gel network is immersed in a 10- to 15-fold higher volume solution of the selected polymer to react with the active surface functional groups of silica for structural strengthening [29]. Another is the one-pot synthesis method, whereby a predetermined amount of the selected polymer is added to the silica sol before gelation occurs, which allows the polymer to react not only with the active surface functional groups of the silica but also inside the bulk part to strengthen the silica gel network [30]. Moreover, the amount of polymer solution used for the synthesis makes a considerable difference to the large-scale production cost of aerogels, considering that the latter method is obviously more efficient for synthesizing hybrid polymer–silica aerogel compared to the post-gelation washing. Thus, preparing hybrid silica aerogel using different organic polymers via a cost-effective one-pot sol–gel synthesis method is an efficient approach [14]. However, enhancing chemical crosslinking with organic polymers causes an interruption in the pore structure of the pristine silica aerogel, which is unfavorable for maintaining a high surface area, low density, and high porosity. It also creates a solid path for heat flow through it, which mainly affects the ultralow thermal conductivity and high thermal stability of silica aerogel and, thereby, limits its applicability.

In this investigation, we reported a one-pot sol–gel synthesis of hybrid poly(acrylamide-co-acrylic acid)-silica aerogel (TGP_X aerogel) via epoxy ring-opening polymerization with enhanced thermal stability and the mechanical properties of pristine silica aerogel. To the best of our knowledge, for the first time, P(AAm-CO-AAc) is used as a crosslinking polymer to enhance the mechanical strength of pristine silica aerogel. In the present study, organic–inorganic hybrid aerogels were prepared by adding P(AAm-CO-AAc) to a tetramethyl orthosilicate (TMOS) silica precursor with (3-glycidyloxypropyl)trimethoxysilane (GPTMS) as a crosslinking agent via epoxy ring-opening polymerization and supercritical alcohol drying. The high thermal stability of P(AAm-CO-AAc) compared to other available organic polymers provides a great advantage for the field of heat insulation [31]. Moreover, the amide and carboxylic functionalities of P(AAm-CO-AAc) allow it to react with the epoxy groups of GPTMS to form covalent bonds via epoxy polymerization, which enhances the structural strength of the hybrid silica aerogel. Furthermore, the effect of the P(AAm-CO-AAc) (PACA) %% variation on the physical, chemical, structural, thermal, and mechanical properties of the hybrid TGP_X (X denotes the wt% of the polymer) aerogels was investigated and reported.

## 2. Results and Discussion

**Scheme 1:**
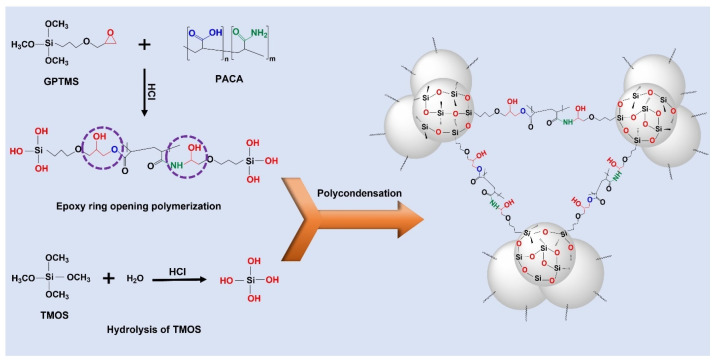
The reaction mechanism in the one-pot sol–gel synthesis of hybrid TGP_X aerogels.

The thermal properties of the hybrid aerogels were investigated by assessing the passage of thermal energy through the material, which occurs via three different mechanisms: solid conductivity, gaseous conductivity, and radiative conductivity [32,33]. When it comes to heat flow through a porous aerogel material, one must consider various factors, such as the porosity, pore size, density, and the type of material used in the synthesis, all of which can significantly impact the heat transfer properties of the aerogel. Thus, carefully analyzing and understanding these factors is crucial for synthetic optimization since, while controlling these parameters, the mechanical properties of aerogels can be deprived. On the other hand, organic–inorganic hybridization generally enhances the mechanical properties, such as the brittleness of an inorganic aerogel, which leads to suppressing the thermal properties of the inorganic aerogel. Hence, to overcome these drawbacks and enhance the mechanical properties of silica aerogel without affecting its thermal properties, we strategically used the highly thermally stable PACA polymer as a crosslinking polymer, which can covalently bond with the silica aerogel, as well as help to control the thermal properties of the silica aerogel. The possible reaction mechanism during the sol–gel synthesis of the TGP_X aerogels via in situ epoxy ring-opening polymerization is illustrated in Scheme 1. Here, epoxy ring-opening polymerization was performed by using the epoxy group of GPTMS and the COOH and CONH_2_ groups of the PACA polymer, where the COOH and CONH_2_ groups acted as a nucleophile and donated a pair of electrons for the epoxy ring-opening polymerization process [34,35], which improves the structural strength of the TGP_X aerogels by forming strong covalent bonds between the GPTMS and PACA polymer. In addition, hydroxyl groups that formed due to the epoxy ring-opening of GPTMS also allowed a further condensation reaction with the hydrolyzed TMOS precursor. In addition to the siloxane bonding, the strong covalent bonds formed by the epoxy ring-opening polymerization between the GPTMS and PACA imparted crack-free hybrid TGP_X aerogel monoliths. Increasing the wt% of the PACA led to complete structural crosslinking and provided improved mechanical strength, as determined by the increase in the compression modulus values of the hybrid TGP_X aerogels compared to the pristine silica aerogel. Unlike that seen in other organic–inorganic hybrid aerogels, the high thermal stability and low thermal conductivity of the PACA polymer can restrict structural heat conduction through the solid part of the TGP_X aerogel, which could maintain the thermal conductivity and, more importantly, enhance the thermal stability compared to pristine silica aerogel. Thus, hybrid TGP_X aerogels were prepared with a variation of PACA (wt%), and their impact on the mechanical and thermal properties was studied.

### 2.1. The Bonding Mechanism of TGP_X Aerogels

#### 2.1.1. Fourier-Transform Infrared (FTIR) Analysis of the TGP_X Aerogels

**Figure 1:**
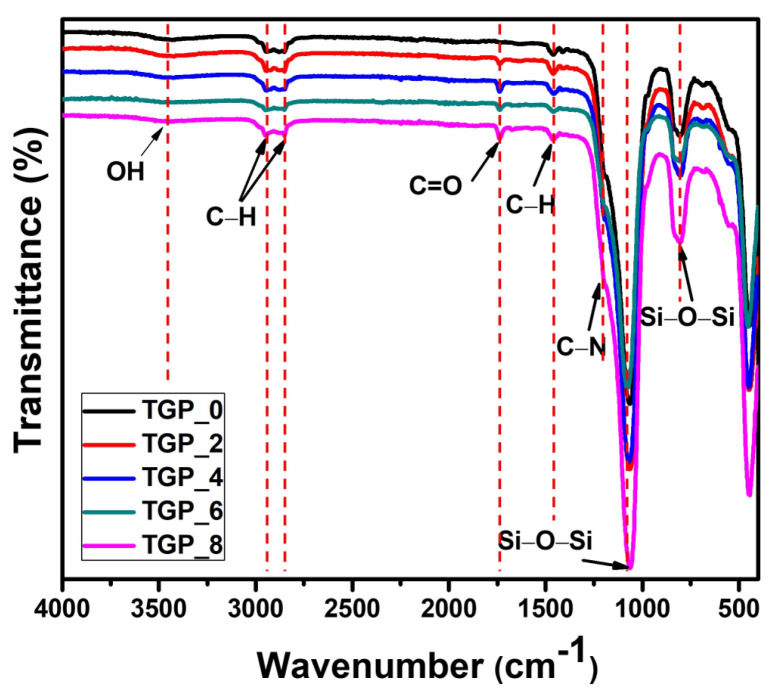
FTIR spectra of the TGP_X aerogels with varying PACA concentrations.

To probe the bonding mechanism of the TGP_X aerogels with the varying PACA polymer’s wt%, FTIR spectra of the TGP_X aerogels were carried out and are depicted in Figure 1. The predominant absorption peaks occurring at 1060, 800, and 445 cm^−1^ are attributed to the Si–O–Si asymmetric and symmetric stretching and deformation vibrations, respectively [36,37,38]. The vibrational peaks at 1400, 2860, and 2946 cm^−1^ correspond to C–H deformation, C–H symmetric stretching, and asymmetric stretching, respectively, arising from the GPTMS and PACA polymers [39]. The vibration peaks at 1736 and 1212 cm^−1^, associated with the C=O and C–N groups, can be seen in the FTIR spectra of all of the samples except for the TGP_0 aerogel sample, which confirms the presence of the PACA polymer. Moreover, the peak intensity of C=O increased with the increasing wt% of PACA in the rest of the hybrid TGP_X samples [40,41]. The FTIR spectrum of the crosslinked hybrid TGP_X aerogels showed a broad and small vibrational peak at around 3400 cm^−1^, corresponding to the –OH group, compared to the TGP_0 aerogel, which further confirms the consumption of formed hydroxyl groups due to the epoxy ring-opening of the GPTMS through the reaction with the TMOS precursor. Thus, the FTIR spectrum of the TGP_X aerogels with the varying PACA polymer’s wt% verify the bonding mechanism of PACA in hybrid TGP_X aerogels.

#### 2.1.2. X-ray Photoelectron Spectroscopy (XPS) Analysis of the TGP_X Aerogels

**Figure 2:**
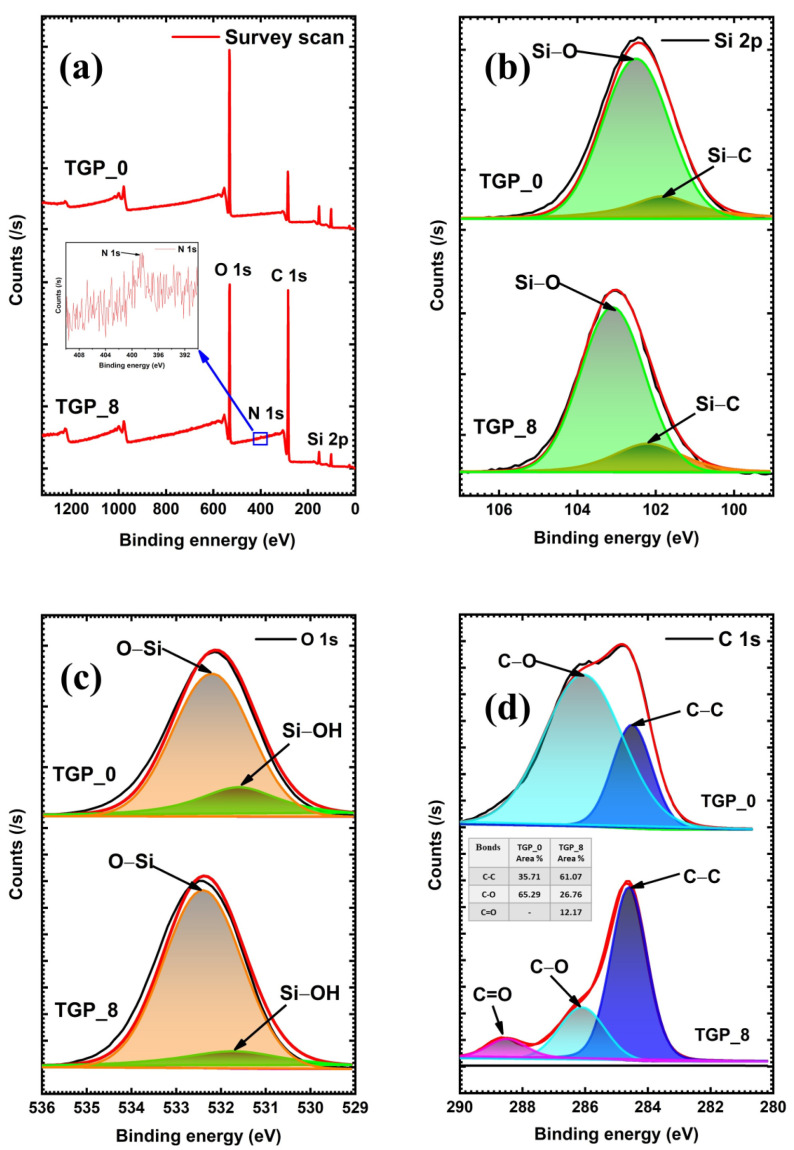
XPS analysis of (**a**) survey scan spectra and high-resolution (**b**) Si 2p, (**c**) O 1s, and (**d**) C 1s spectra of the TGP_0 and hybrid TGP_8 aerogels.

The XPS spectra showcased in Figure 2 offers significant insight into the surface elemental composition of the TGP_0 and TGP_8 aerogels, which is crucial for understanding the epoxy ring-opening polymerization process. The XPS survey scan spectra confirm the presence of Si, C, N, and O elements in the TGP_0 and TGP_8 aerogels (Figure 2a). The high-resolution XPS of the Si 2p spectra in Figure 2b exhibits distinct peaks at 101.8 and 102.5 eV for the TGP_0 aerogel and 101.9 and 103.3 eV for the TGP_8 aerogel, corresponding to the Si-C and Si-O bonds, respectively, where the Si–O bonds indicate the presence of a (IV) chemical state [42,43]. Likewise, Figure 2c provides a clear visualization of the high-resolution O 1s spectra, revealing distinct peaks of the XPS peak for the TGP_0 aerogels located at 531.5 and 532.2 eV, which confirm the presence of Si–OH and O–Si bonds. Similarly, for the TGP_8 aerogel, the peaks at 531.7 eV and 532.4 eV are indicative of Si–OH and O–Si bonding, respectively [44,45]. It is worth noting that these spectra were obtained from both the GPTMS and TMOS precursors, providing substantial evidence to support the presence of Si–O–Si siloxane bonding in hybrid TGP_8 aerogels. Furthermore, the high-resolution XPS spectrum of the C 1s for the TGP_0 aerogel in Figure 2d is deconvoluted into two peaks at 284.5 eV and 286.1 eV, corresponding to the C–C and C–O bonds, respectively, with a measured peak area component ratio of 1:2 for C–C:C–O, where the C–O bonds confirm that the TGP_0 aerogel had undergone epoxy ring surface modification [46]. Comparatively, the high-resolution XPS of the C 1s spectrum of the TGP_8 aerogel sample deconvoluted into three peaks at 284.5, 286.2, and 288.5 eV, associated with C–C, C–O, and C=O bonds, respectively, with a measured peak area component ratio for the C–C:C–O:C=O of 6:3:1, as tabulated in the inset of Figure 2d [47,48,49]. A significant increase in the peak area for the C–C bonds and a profound decrease in the peak area of the C–O bonds are indicative of the epoxy ring-opening polymerization between the GPTMS and PACA. Furthermore, the presence of C=O bonds and N atoms (the inset in Figure 2a) from PACA confirmed the use of the COOH and CONH_2_ groups as nucleophiles in the epoxy ring-opening reaction [48]. Therefore, these observations from the XPS analysis results confirm the successful formation of hybrid TGP_X aerogels via in situ epoxy ring-opening polymerization.

### 2.2. Physical Property and Textural Analysis of the TGP_X Aerogels

Thermal conductivity and thermal stability are the two most crucial parameters to assess the thermal insulation properties of an aerogel, while the thermal conductivity and stability of porous aerogels are principally subjected to heat transfer through the solid phase, gas phase, and radiation, which are mainly influenced by the density and porosity of the material [50]. Therefore, the physical properties of hybrid TGP_X aerogels with varying PACA polymers (a 0 to 8 wt%) in terms of density, porosity, thermal conductivity, specific surface area, pore volume, and average pore diameter are measured and summarized in Table 1. As expected, very small and monotonous increases in the bulk density and thermal conductivity values of the TGP_X crosslinked aerogels were observed with the increasing PACA concentration, wherein the bulk densities of the TGP_X crosslinked aerogels increased from 0.084 to 0.12 cm^3^/g due to the addition of the molecularly dense PACA polymer. While the porosity percentage of the TGP_X aerogel, calculated by using Equation (1), decreased from 95.57% to 93.68%:

Porosity (%) = [1 − (ρb/ρs)] × 100, (1)

where ρb is the bulk density, and ρs is the skeletal density (~1.9 g/cc) [38]. This predictable decrease in porosity is due to the interruption in the pore structure of the TGP_X aerogel caused by crosslinking. Moreover, the increase in thermal conductivity from 22.4 to 30.6 mW/mK can be attributed to crosslinking between the GPTMS and PACA polymer, which generates heat transfer passages due to the increase in density and decrease in porosity with the increasing PACA concentration [51]. In general, the addition of organic content to silica aerogel leads to an increase in thermal conductivity. For example, Ghica et al. [52] observed an increase in thermal conductivity from 31.3 to 85.4 mW/mK after the reinforcement of a silica aerogel with optimized polyamide pulps. Moreover, Merillas et al. [53] analyzed that the synthesized polyurethane-reinforced silica composite aerogel exhibited enhanced thermal conductivity from 33.4 to 52.5 mW/mK. Similarly, Ilhan et al. [54] and Hae-Noo-Ree Jung et al. [55] reported improved thermal conductivity of hydrophobic silica aerogels to around 41 and 131 mW/mK with the addition of polystyrene and poly(methyl methacrylate), respectively. These results confirm that the addition of an organic polymer drastically increases the thermal conductivity of hybrid aerogels. Hence, the thermal conductivity values of the TGP_X aerogels in the present work from 22.4 to 30.6 mW/mK are comparable to those of these previously reported organic–inorganic hybrid aerogels [38,52,53,54,55], owing to the synergy among the thermally less conductive PACA polymer (60 mW/mK) [56] and the silica aerogel.

**Figure 3:**
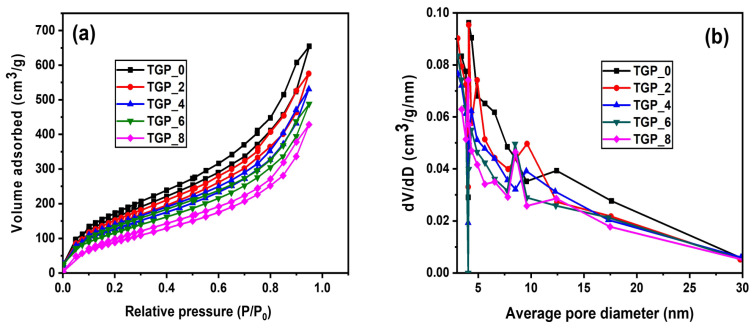
(**a**) Nitrogen adsorption–desorption isotherms and (**b**) the average pore size distribution of the TGP_X aerogels with varying PACA concentrations.

**Figure 4:**
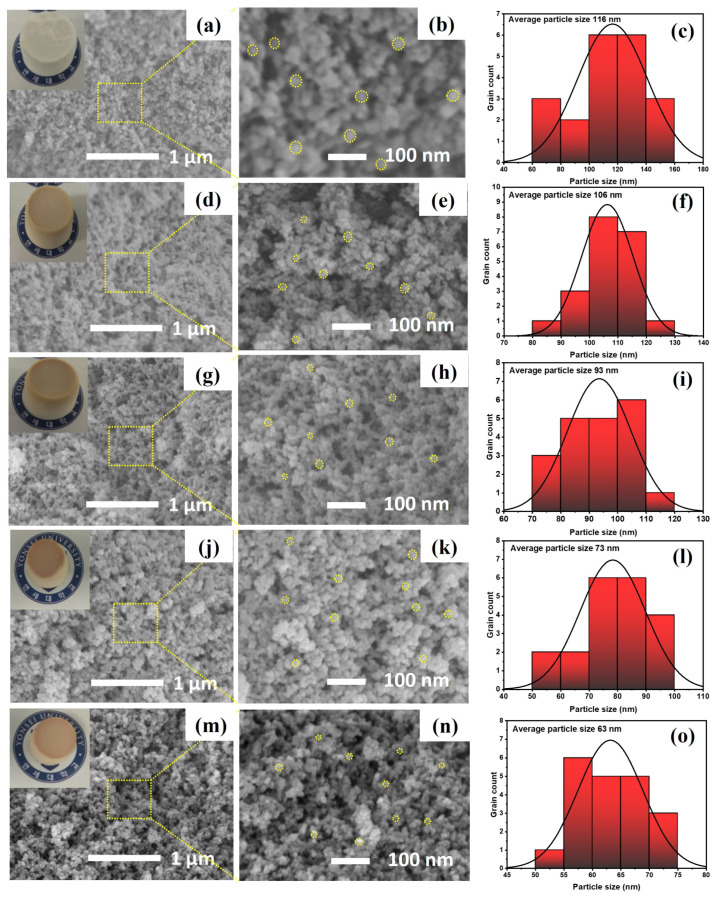
FESEM images with inset photographs and particle distribution plots of the TGP_X aerogels with varying PACA concentrations: (**a**–**c**) TGP_0, (**d**–**f**) TGP_2, (**g**–**i**) TGP_4, (**j**–**l**) TGP_6, and (**m**–**o**) TGP_8 aerogels.

Nitrogen adsorption–desorption isotherms were obtained for the TGP_X aerogels; the corresponding Brunauer–Emmett–Teller (BET) and Barrette–Joyner–Halenda (BJH) plots for a specific surface area and pore size distribution are shown at Figure 3a and Figure 4b, respectively. All the isotherms shown in Figure 3a belong to type IV curves and H3-type hysteresis, indicating a mesoporous structure of the TGP_X aerogels. The specific surface area of the TGP_X aerogel decreased bit-by-bit from 608.6 to 369.0 m^2^/g with the increasing PACA polymer’s wt%. Correspondingly, the pore volume decreased from 1.01 to 0.60 cm^3^/g, as obtained from the BJH pore size distribution curves shown in Figure 3b. Moreover, the average pore diameters were found in the range of 6.44–7.38 nm, which confirms the mesoporous structure of the TGP_X aerogels. The results above clearly demonstrate that the specific surface area and pore volume decrease with an increase in the wt% of the PACA polymer in the TGP_X aerogel. Thus, it is evident that the epoxy crosslinking polymerization induces the shrinkage and deformation of the pore size, which resulted in a decrease in the specific surface area and pore volume of the hybrid TGP_X aerogels.

### 2.3. Morphological Analysis of the TGP_X Aerogels

The surface morphologies and reduction in the average particle sizes of the TGP_X aerogels with the PACA polymer’s wt% were observed through FESEM analysis, as shown in Figure 4a–o. Figure 4a,b show FESEM images of the TGP_0 aerogel at different magnifications, which clearly exhibit aggregated secondary silica particles having an average particle size of ~116 nm (Figure 4c) with a highly porous structure. However, the FESEM images and histograms of the average particle size distribution of the hybrid TGP_X aerogels with varying PACA polymer concentrations (a 2 to 8 wt%) shown in Figure 4d–o demonstrate interconnected silica particles forming a uniform mesoporous network with the decreasing average particle size. Moreover, the observed decrease in the average particle size from 116 nm (TGP_0) to 63 nm (TGP_8) with the increasing PACA concentrations was owed to constraints induced by the PACA on the growth of the silica particles. Increased epoxy polymer crosslinking with the increasing PACA polymer wt% reinforces the structural strength of the hybrid TGP_X aerogels and forms crack-free and robust silica aerogels (the images in the insets of Figure 4a,d,g,j,m). In addition, the uniform mesoporous distribution provides a tortuous path for heat conduction, which effectively restricts heat transfer through the aerogel, thereby successfully bestowing the thermal insulating capability of the hybrid TGP_X aerogel successfully.

### 2.4. Mechanical Property Analysis of the TGP_X Aerogels

**Figure 5:**
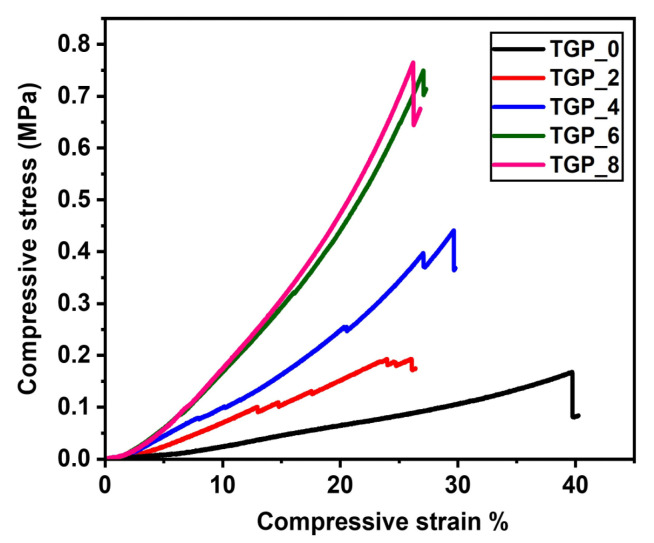
The compressive stress–strain curve of the TGP_X aerogels with varying PACA concentrations.

The industrial applicability of silica aerogel recommends that it should be mechanically strong enough and hold out against large compressive loads. However, synthesizing hybrid silica aerogel is one of the most effective approaches to obtaining mechanically strong aerogel, which makes it able to survive with a higher compressive load. The mechanical properties of the TGP_X aerogels were measured by using a compressive strength measurement (Shimadzu, Japan, AGX-50kNVD) under the compressive load. The compression stress–strain curves of the TGP_X aerogels with varying PACA concentrations are shown in Figure 5 to investigate the mechanical properties. The compression stress of the TGP_X aerogels increases linearly from 0.16 to 0.76 MPa with increased crosslinking polymerization between the GPTMS and PACA concentrations compared to the pristine TGP_0 aerogel. Hence, the compression modulus of the TGP_X aerogels increased from 0.4 MPa for the pristine TGP_0 aerogel to 2.9 MPa for the PACA hybridized TGP_8 aerogel (Table 2). It is observed that the compression modulus of the TGP_X aerogels enhanced with increasing the PACA concentration due to the augmented epoxy ring-opening polymerization. Moreover, a minute increase in compression stress led to minimal changes in the compression modulus of the TGP_6 and TGP_8 aerogels, which suggests saturation and completed epoxy ring-opening polymerization in the hybrid TGP_X aerogels.

### 2.5. The Thermal Stability and Performance of the TGP_X Aerogels

**Figure 6:**
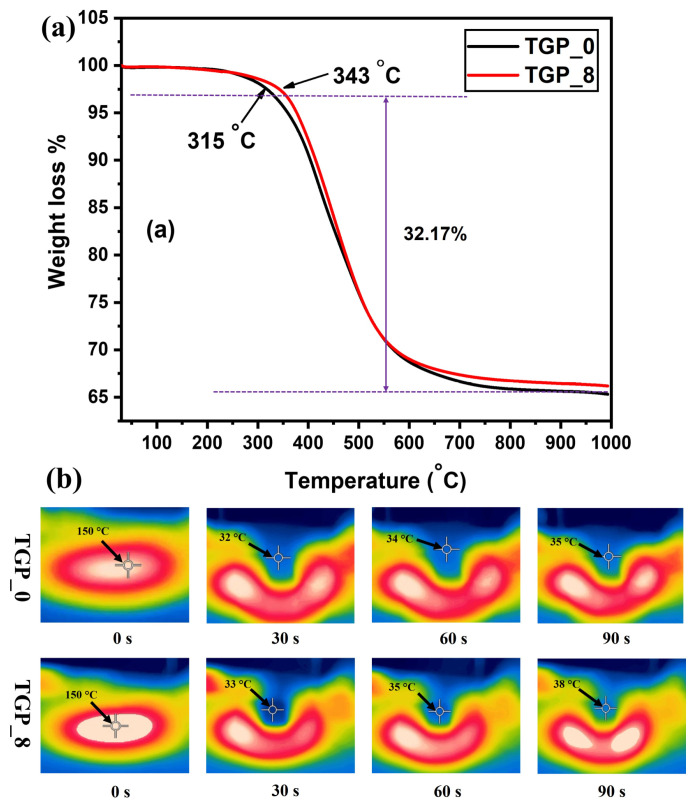
(**a**) Thermogravimetric analysis (TGA) plots and (**b**) thermal insulation performances of the pristine TGP_0 and hybrid TGP_8 aerogels: the IR images of the pristine TGP_0 and hybrid TGP_8 aerogels on a hot plate at a temperature of 150 °C viewed from the side.

The thermal stability of TGP_X aerogels was studied using thermogravimetric analysis (TGA) for pristine TGP_0 and hybrid TGP_8 aerogels from 30 to 1000 °C in a nitrogen atmosphere with a heating rate of 10 °C min^−1^ for practical insulating applications. The comparative TGA plotting for the thermal stability of the pristine TGP_0 and hybrid TGP_8 aerogels is shown in Figure 6a. A small initial weight loss (~2.5%) occurred with increasing the temperature up to 120 °C, which can be attributed to the evaporation of water molecules, moisture, and persistent organic solvent in the pores of the aerogels. Further, major weight loss was observed in the second stage, from 315 to 800 °C, due to the decomposition of methyl groups and organic content from the GPTMS and PACA polymer in the TGP_X aerogel. Moreover, second-stage decomposition commenced at 315 and 343 °C for the pristine TGP_0 and hybrid TGP_8 aerogels, respectively, which indicates that the thermal stability of the hybrid TGP_X aerogel increased with the addition of the thermally stable PACA polymer [57]. Furthermore, when the temperature was raised to 1000 °C, a minimal weight loss difference of 1% between the pristine TGP_0 and hybrid TGP_8 aerogels was observed due to the decomposed polymer residue. Therefore, the above results endorse that the hybrid TGP_8 aerogel has higher thermal stability than the pristine TGP_0 aerogel, which can be ascribed to the high thermal stability of the silica aerogel and PACA polymer, as well as the presence of strong covalent bonding between the GPTMS and PACA via epoxy ring-opening polymerization.

To verify the progressive time-dependent thermal insulating performance of the pristine TGP_0 aerogel (a diameter of 20 mm and a thickness of 21 mm) and hybrid TGP_8 aerogel (a diameter of 18 mm and a thickness of 19 mm), samples were subjected to a hot plate heated at ~150 °C, and time-dependent IR thermal images were captured, as shown in Figure 6b. In the beginning, the pristine TGP_0 aerogel proved its exceptional thermal insulation capability by maintaining a temperature below 35 °C, even after being placed on a hot plate for 90 s. Similarly, the hybrid TGP_8 aerogel placed on a hot plate also showed excellent thermal insulation performance by keeping the temperature below 38 °C when the time passed for 90 s. Thus, these findings illustrate the remarkably similar thermal insulation performances of the pristine TGP_0 and hybrid TGP_8 aerogels, which is attributed to their low thermal conductivity and high thermal stability stemming from the unique mesoporous network structure and hybridization with the thermally stable PACA polymer of the TGP_X aerogels, respectively.

**Figure 7:**
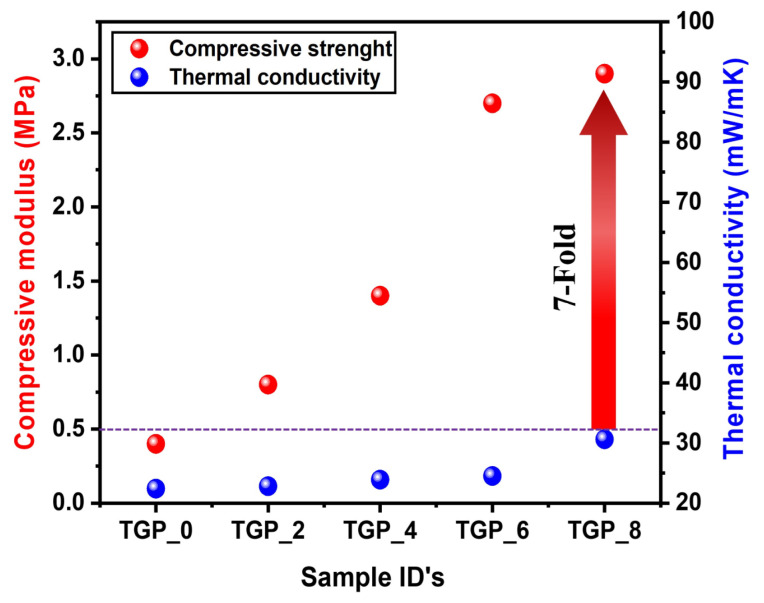
A combined plot of the compression modulus and thermal conductivity of the TGP_X aerogels with varying PACA concentrations.

The detailed comparison of the reported hybrid polymer–silica aerogel is summarized in Table 3 in terms of the reinforcing method, density, specific surface area, thermal conductivity, thermal stability, and compression modulus. In comparison with the previous reports, it is observed that the hybrid polymer–silica aerogel shows a higher compression modulus by sacrificing the prime properties, such as the bulk density, specific surface area, and, most importantly, thermal conductivity, owing to the crosslinking density [23,58,59]. On the other hand, despite maintaining the aforementioned prime properties, the mechanical properties of the hybrid silica aerogel deteriorated [13,27,47,53,60]. However, in the present work, the hybridized TGP_X aerogels demonstrated better thermal properties, as well as an outstanding seven-fold increase in the compression modulus compared to the pristine TGP_0 aerogel while maintaining their thermal conductivity values below 30 mW/mK (Figure 7). Such exceptional outcomes of the hybrid TGP_X aerogels are credited to the complete hybridization between the GPTMS and PACA, enhancing the compressive strength of the silica aerogel, which validates the structural reinforcement of the hybrid TGP_X aerogels. Meanwhile, the high thermal stability and low thermal conductivity of the PACA polymer reward the enhancing thermal stability while maintaining the low thermal conductivity of the hybrid TGP_X aerogels. In addition, the fundamental properties of the pristine TGP_0 aerogel, such as the density, porosity, and high specific surface area, were efficiently maintained in the hybridized TGP_X aerogels. Thus, the selection of PACA as a reinforcing organic polymer is an important part of synthesizing hybridized TGP_X aerogels, which show an outstanding improvement in the mechanical and thermal properties of silica aerogels, which are more beneficial for enhancing its thermal insulation applicability.

## 3. Conclusions

Thermally stable and structurally reinforced hybrid TGP_X aerogels were successfully prepared via a facile one-pot sol–gel synthesis method by exploiting epoxy ring-opening polymerization using GPTMS and PACA as the crosslinker and reinforcing polymer, respectively. The specific surface area of the TGP_X aerogels decreased from 608.6 to 369.0 cm^3^/g with the increasing polymer content in the aerogel, while their porosity and density values were maintained between 95.57% to 93.68% and 0.084 to 0.12 cm^3^/g, respectively. Furthermore, the in situ epoxy crosslinking of pristine silica aerogel with the PACA polymer reveals outstanding enhancement in the compression modulus, from 0.4 to 2.9 MPa of the TGP_X aerogels, while maintaining a thermal conductivity from 22.4 to 30.6 mW/mK. In addition, TGA analysis confirms the increase in thermal stability of the hybrid TGP_X aerogel from 315 to 343 °C. These results suggest that the epoxy crosslinking between the GPTMS and PACA polymer forms strong covalent bonding that improves the mechanical, thermal, and textural properties of TGP_X aerogels. Conclusively, the facile one-pot synthesis of highly robust and thermally insulating TGP_X aerogel via epoxy ring-opening polymerization using PACA paves a novel way for preparing hybrid aerogels for industrial thermal insulation applications.

## 4. Materials and Methods

### 4.1. Materials

The synthesis of the hybrid TGP_X aerogels was carried out by using TMOS (Si(OCH_3_)_4_; 98%) as a silica precursor, GPTMS (C_9_H_20_O_5_Si; 98%) as a crosslinker, P(AAm-CO-AAc)) (PACA) as a reinforcing polymer, ammonium fluoride (NH_4_F) as a base catalyst, and HCl as an acidic catalyst, all of which were purchased from Sigma-Aldrich (St. Louis, MO, USA). Methanol (Duksan Chemicals, Ansan, Republic of Korea) was used as a solvent, and deionized (DI) water was used in the experiments. All the chemicals were used as received without any further purification.

### 4.2. Synthesis of the TGP_X Aerogels

**Figure 8:**
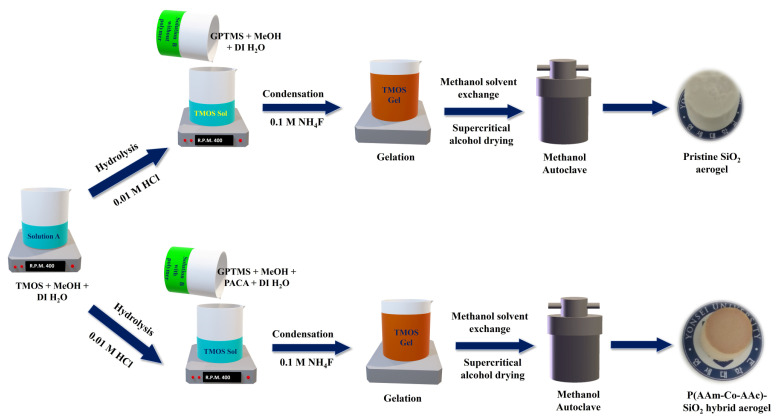
An illustration of one-pot sol–gel synthesis of the pristine TGP_0 and hybrid TGP_X aerogels.

TGP_X aerogels were prepared by using a facile one-pot sol–gel synthesis method, as schematically represented in Figure 8, which consists of pristine TGP and hybrid TPG_X aerogel, explained as follows: Initially, solution A was prepared using a mixture of (0.7 mL) TMOS, (3 mL) methanol, and (0.1 mL) DI water, to which (0.5 mL) optimized 0.01M HCl was added dropwise as the acidic catalyst for hydrolysis, followed by 1 h of stirring at room temperature. Meanwhile, PACA polymer solutions were prepared with different wt% of the polymer (2, 4, 6, and 8 wt%) in DI water. Subsequently, solution B was prepared by using a mixture of (0.3 mL) GPTMS, (3 mL) methanol, (0.1 mL) DI water, and (0.5 mL) PACA polymer solution with different wt%, followed by stirring for 4 h at room temperature. Then, for hybrid sol–gel synthesis, solution B was added to solution A, followed by vigorously stirring for 1 h. After allowing an extra 2 h of hydrolysis, (0.5 mL) 0.1M of NH_4_F was added as the basic catalyst for condensation, followed by 10 min of stirring. For all samples, the gel usually formed within 30 min at 55 °C with negligible shrinkage. The TGP_X gels were aged in an oven for 3 h at 55 °C for further completion of the condensation reaction and to strengthen the gel structure. Monolithic aerogels were obtained through methanol solvent exchange and supercritical alcohol drying (SAD) in Parr autoclave (Moline, IL, USA) at 265 °C and 120 bar pressure for 4 h. The preparation of hybrid TGP_X aerogels was performed by using a TMOS:GPTMS:methanol:DI water molar ratio of 1:0.3:30:2, with varying the wt% of the PACA polymer solution as 0, 2, 4, 6, and 8 wt%, depending upon the solubility of the polymer in the methanol. The influence of the varying PACA polymer (wt%) on the structural, morphological, and chemical properties and their impact on mechanical strength and thermal stability of TGP_X aerogels was investigated using different types of characterization.

### 4.3. Characterization of the TGP_X Aerogels

The structural, morphological, chemical, and physical properties of the prepared TGP_X aerogels were investigated by using various characterization techniques. The surface functional group of TGP_X aerogels was examined by using FTIR spectroscopy (PerkinElmer 1760X spectrometer, Waltham, MA, USA) over a wavelength range from 400 to 4000 cm^−1^. Chemical crosslinking between GPTMS, PACA, and TMOS was confirmed by using X-ray photoelectron spectroscopy (XPS: K-Alpha, Thermo Fisher Scientific, Darford, UK) with a monochromatic Al X-ray source (Al Kα line: 1486.6 eV, 3 mA, and 12 kV). The surface morphologies of the TGP_X aerogels were investigated via field emission scanning electron microscopy (FESEM; JEOL JSM-7001F, Tokyo, Japan) performed at 5–10 kV. Surface area, pore volume, and pore size of TGP_X aerogels were calculated by using the N_2_ adsorption–desorption isotherm data obtained from Brunauer–Emmett–Teller (BET) and Brunauer–Emmett–Teller (BJH) analyses (Quantachrome Instruments v10.0). According to previous reports, the bulk density (ρb) of TGP_X aerogel samples was calculated using the mass-to-volume ratio. The porosity (%) of the TGP_X aerogels was calculated from the reported Formula (1) by using ρs as the skeletal density (~1.9 g/cc) of TGP_X aerogel [38].

The thermal conductivity of TGP_X aerogels was measured by using an ASTM D7984 (Trident-MTPS, C-Therm, Fredericton, NB, Canada). Thermogravimetric analysis (TGA: SDT-2790, TA Inc., Boston, MA, USA) was performed to determine the thermal stability temperature of TGP_X aerogel, where measurements were carried out in the temperature range of 30 °C to 1000 °C with a heating rate of 10 °C min^−1^. The mechanical properties of TGP_X aerogels were measured using compressive strength measurements (Shimadzu, Tokyo, Japan, AGX-50kNVD).

## Figures and Tables

**Table 1:** The physical properties of the TGP_X aerogels with varying PACA concentrations.

Aerogel	Density (g/cm^3^)	Porosity (%)	Thermal Conductivity (mW/mK)	*S_BET_* (m^2^/g)	Pore Volume (cm^3^/g)	Average Pore Diameter (nm)
TGP_0	0.084	95.57	22.4	608.6	1.01	6.65
TGP_2	0.087	95.42	22.8	552.4	0.89	6.44
TGP_4	0.096	94.94	23.9	493.9	0.82	6.65
TGP_6	0.10	94.73	24.5	456.8	0.75	6.60
TGP_8	0.12	93.68	30.6	369.0	0.60	7.38

**Table 2:** The mechanical properties of the TGP_X aerogels with varying PACA concentrations.

Aerogel	Breaking Load at 25% Strain (MPa)	Compressive Modulus (MPa)
TGP_0	0.08	0.4
TGP_2	0.19	0.8
TGP_4	0.36	1.4
TGP_6	0.69	2.7
TGP_8	0.75	2.9

**Table 3:** Comparison of physical properties of TGP_X aerogel and other hybrid polymer–silica aerogels.

Hybrid Silica Aerogels	Reinforcing Method	Density (g/cm^3^)	Specific Surface Area (m^2^/g)	Thermal Conductivity (mW/mK)	Thermal Stability (°C)	Compression Modulus (MPa)	Ref.
TMOS/polyurea	Post-gelation washing	0.47	261	41	300	129	[58]
TEOS/PTPES	Radical polymerization	0.024–0.096	420.5–975.7	-	190	0.03–0.062	[47]
TMOS/BTESB/BTMSH/TMSPM	One-pot synthesis	0.13–0.39	7.0–719.0	39–93	392–518	0.0017–0.0067	[13]
TEOS/APTES/BTMSH/BPGE/RGE	Post-gelation washing	0.05–0.35	99–375	-	-	1.4–97.8	[23]
TEOS/polyimide	Chemical imidization	0.09–1.21	210.1–272.1	23–24	527.9	0.046–0.112	[59]
TMOS/polyacrylate	One-pot synthesis	0.24–0.52	53–155.1	65–138	-	0.786–2.65	[27]
TEOS/polyureathane	One-pot synthesis	0.79–3.91	760.2–912.8	33.2–71.3	-	0.13–0.45	[60]
TMOS/polyurea	Post-gelation washing	0.18–0.61	140–969	-	350	0.01–4	[53]
PACA-silica aerogel	One-pot synthesis	0.084–0.12	369.0–608.6	22.4–30.6	343	0.4–2.9	This work

## Data Availability

Data will be made available on request.
